# Deep Network for the Automatic Segmentation and Quantification of Intracranial Hemorrhage on CT

**DOI:** 10.3389/fnins.2020.541817

**Published:** 2021-01-11

**Authors:** Jun Xu, Rongguo Zhang, Zijian Zhou, Chunxue Wu, Qiang Gong, Huiling Zhang, Shuang Wu, Gang Wu, Yufeng Deng, Chen Xia, Jun Ma

**Affiliations:** ^1^Department of Radiology, QingPu Branch of Zhongshan Hospital Affiliated to Fudan University, Shanghai, China; ^2^Beijing Infervision Technology Co., Ltd., Beijing, China; ^3^Department of Neuroradiology, Beijing Tiantan Hospital, Capital Medical University, Beijing, China

**Keywords:** CT, deep learning, intracranial hemorrhage, segmentation, quantification

## Abstract

**Background:**

The ABC/2 method is usually applied to evaluate intracerebral hemorrhage (ICH) volume on computed tomography (CT), although it might be inaccurate and not applicable in estimating extradural or subdural hemorrhage (EDH, SDH) volume due to their irregular hematoma shapes. This study aimed to evaluate deep framework optimized for the segmentation and quantification of ICH, EDH, and SDH.

**Methods:**

The training datasets were 3,000 images retrospectively collected from a collaborating hospital (Hospital A) and segmented by the Dense U-Net framework. Three experienced radiologists determined the ground truth by marking the pixels as hemorrhage area. We utilized the Dice and intra-class correlation coefficients (ICC) to test the reliability of the ground truth. Moreover, the testing datasets consisted of 211 images (internal test) from Hospital A, and 86 ICH images (external test) from another hospital (Hospital B). In this study, we chose scatter plots, ICC, and Pearson correlation coefficients (PCC) with ground truth to evaluate the performance of the deep framework. Furthermore, to validate the effectiveness of the deep framework, we did a comparative analysis of the hemorrhage volume estimation between the deep model and the ABC/2 method.

**Results:**

The high Dice (0.89–0.95) and ICC (0.985–0.997) showed the consistency of the manual segmentations among the radiologists and the reliability of the ground truth. For the internal test, the Dice coefficients of ICH, EDH, and SDH were 0.90 ± 0.06, 0.88 ± 0.12, and 0.82 ± 0.16, respectively. For the external test, the segmentation Dice was 0.86 ± 0.09. Comparatively, the ICC and PCC of ICH volume estimations were 0.99 performed by Dense U-Net that overmatched the ABC/2 method.

**Conclusion:**

This study revealed the excellent performance of hematoma segmentation and volume evaluation based on Dense U-Net, which indicated our deep framework might contribute to efficiently developing treatment strategies for intracranial hemorrhage in clinics.

## Introduction

Intracranial hemorrhages, such as ICH, EDH, SDH, and subarachnoid hemorrhages (SAH), are dangerous with high mortality and low functional recovery rates (Xi et al., 2006; Qureshi et al., 2009; Soffar, 2012). Moreover, the controversy over surgical intervention and conservative management still exists even though there is no significant outcome difference between the treatments (Mayer and Rincon, 2005; Mendelow et al., 2005; Mendelow et al., 2013). Therefore, the estimation of hemorrhage volume plays a critical role among the prognosis parameters to predict the outcome and standardize the clinical treatment (Broderick et al., 1993; Hemphill et al., 2001). Generally, the ABC/2 method has been used to calculate the hematoma volume at the time of symptomatic ICH diagnosis in previous studies (Kothari et al., 1996; Divani et al., 2011; Yaghi et al., 2015). With this approach, the hematoma was estimated as an ellipsoid, and A, B, and C were the orthogonal axes measured from CT or magnetic resonance imaging (MRI) (Hemphill and Lam, 2017; Liu et al., 2019). However, it was difficult to precisely quantify hemorrhage volume due to the limitations of the conventional ABC/2 method. Though the ABC/2 method might be practical and time-efficient for ICH with a single bleeding site, it might lead to inaccurate measurements for ICH with multiple bleeding sites (Huttner et al., 2006), and is not applicable for other intracranial hemorrhage types due to their irregular hematoma shapes. Moreover, a manual delineation of the contours of the hematoma could be time-consuming, which is not suitable for emergency settings.

Therefore, computer-aided image segmentation could provide accurate and fast volume estimation for brain hemorrhages. A recent study showed that the ICH volume could be estimated using a random-forest based machine learning algorithm, with a Pearson correlation coefficient of 0.96 against the manual segmentation (Scherer et al., 2016). However, the study comprised 58 cases in total and was limited to spontaneous ICH. In addition, its accuracy decreased as the hematoma volume increased. Another study demonstrated that region proposal convolution neural networks could be used to simultaneously detect and segment brain hemorrhages (Chang et al., 2018). However, the dataset used in this study was from a single institution, and the ground truth was generated semi-automatically and was determined by only one radiologist, which might not be reliable for training and testing of the deep learning (DL) model.

In this study, we firstly applied a DL model based on Dense U-Net (Ronneberger et al., 2015; Guan et al., 2019) architecture to segment and quantify three types of brain hemorrhage (ICH, EDH, and SDH) on non-contrast CT images. Since contrast CT scans are needed for accurate SAH segmentation, SAH was excluded from the current study. To test the reliability of the ground truth masks from the experienced radiologists utilized in this study, we calculated the Dice and ICC among the three experienced radiologists. Then, on the internal and external test set, the segmentation Dice was evaluated to test whether the constructed model based on Dense U-Net could be utilized to segment the intracranial hemorrhage from the head CT successfully. Finally, we did a comparative analysis on ICC and PCC of ICH volume estimations between the deep model and the ABC/2 method to validate the effectiveness of the constructed framework based on Dense U-Net. We hope that this study could assess the feasibility of the constructed deep model for the accurate segmentation and quantification of intracranial hemorrhage on non-contrast CT, and provide a guide for clinical decision-making.

## Materials and Methods

### Data Collection

Institutional Review Board (IRB) approval was received from collaborating hospitals and informed consent was waived for this retrospective study. A total of 3,000 non-contrast brain CT scans containing ICH, EDH, and SDH were retrospectively collected from Beijing Tiantan Hospital Affiliated to Capital Medical University (Hospital A) for model training and validation. Each hemorrhage type had 1,000 scans, which were randomly partitioned for model training and validation with a ratio of 80:20%. Another 211 scans from the same hospital were reserved as the internal test set, where ICH, EDH, and SDH had 61, 87, and 63 scans, respectively. To test the validity of the deep framework, we collected 86 ICH CT scans from the QingPu Branch of Zhongshan Hospital Affiliated to Fudan University (Hospital B) as an independent testing set (i.e., external test set). Besides, in order to evaluate the performance of the model on non-hemorrhagic cases, 450 cases containing 48 hemorrhagic and 402 non-hemorrhagic cases were also collected from Hospital B. All images were acquired using brain CT protocols on scanners from various vendors, with the x-ray tube voltage around 120 kV and current around 400 mA. The matrix size was 512 × 512 and most of the scans had a slice thickness of 5 mm.

### Ground Truth Determination

To determine the segmentation ground truth for model training, each CT scan was independently examined by three certified neuroradiologists and annotated using the 3D Slicer (Carrboro, NC, United States) (Kikinis et al., 2014). The common segmentations, i.e., pixels that were marked as hemorrhage positive by at least two neuroradiologists, were considered as the ground truth and each annotated slice was inputted into the 2D segmentation network for training. The segmentation by the radiologists against the ground truth and their volume estimation agreements were also evaluated using the Dice and ICC, respectively.

### Model Construction and Training

**FIGURE 1:**
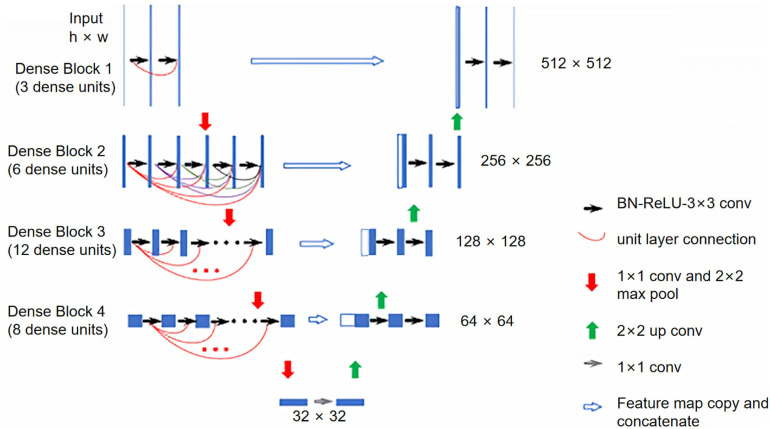
Illustration of the constructed Dense U-Net. The Dense U-Net had four dense blocks, with 3, 6, 12, and 8 dense units in each dense block. The growth rate was 32 for all dense units. BN, batch normalization; ReLU, rectified linear unit; Conv, convolution.

The model was constructed on the MXNet platform (Chen et al., 2015). The DenseNet encoder consisted of 4 dense blocks, each followed by a transition layer (Huang et al., 2017). Each dense block had 3, 6, 12, and 8 dense units, respectively and each dense unit had a growth rate of 32. Feature maps extracted from each dense block were concatenated with the up-sampled maps to form a U-Net structure. The input into the model was one CT scan, and the output was the segmented masks for all slices. Detailed structures of the constructed model are illustrated in Figure 1.

During the implementation of the DL model, the He-normal initialization was used for all convolutional kernels (He et al., 2015). The DL model was trained using a binary cross-entropy loss function (De Boer et al., 2005) which can make the training more stable than using a Dice loss function and was improved with a stochastic gradient decay (SGD) optimizer (Bottou, 2010). The learning rate was set to 0.001 with a momentum of 0.99. The model was trained on four Nvidia GTX 1080 graphic processing units (32 gigabytes total memory capacity) with a batch size of one. No image augmentation was applied during the model training. The training process was finished after 20 epochs when the validation loss had no improvement. No dropout layers were used without an overfitting problem (Srivastava et al., 2014). With the DL model, the pixel-wise segmentation results were achieved. Combining with CT thickness, we could calculate the volume of each pixel. The hemorrhage volume was calculated by accumulating all the volumes of pixels in the hemorrhage region.

### Performance and Statistical Analysis

After training, the model was tested on the reserved testing data. The segmentation performance of the model was analyzed using the Dice coefficient against the ground truth. The sum of the segmented areas of each slice was multiplied by the corresponding slice thickness, yielding the hemorrhage volume of a patient. The segmentation-based volume estimation was then compared with the ground truth using scatter plots, ICC, and PCC. A non-parametric Wilcoxon signed-rank test (Rey and Neuhäuser, 2011) was used to evaluate the systematical volume bias of the segmentation-based method due to the non-normal distribution of the hemorrhage volume difference.

## Results

### Manual and Automatic Segmentation Evaluation

For the testing data originating from Hospital A, Table 1 demonstrates the performance of the segmentation by the radiologists against the ground truth and their in-group volume estimation agreements using Dice and ICC, respectively. The high Dice (0.89–0.95) and ICC scores (0.985–0.997) showed that the manual segmentations were consistent among the three radiologists and the ground truth was reliable for model training and evaluation.

**TABLE 1:** Dice and ICC of the manual segmentation and volume measurement.

Hemorrhage type	Dice			ICC
	Radiologist 1	Radiologist 2	Radiologist 3	
ICH	0.95 ± 0.03	0.90 ± 0.13	0.93 ± 0.04	0.9963
EDH	0.93 ± 0.11	0.93 ± 0.09	0.93 ± 0.08	0.9967
SDH	0.91 ± 0.11	0.89 ± 0.09	0.93 ± 0.05	0.9847

*ICC, intra-class correlation coefficient; ICH, intracerebral hemorrhage; EDH, extradural hemorrhage; SDH, subdural hemorrhage.*

**FIGURE 2:**
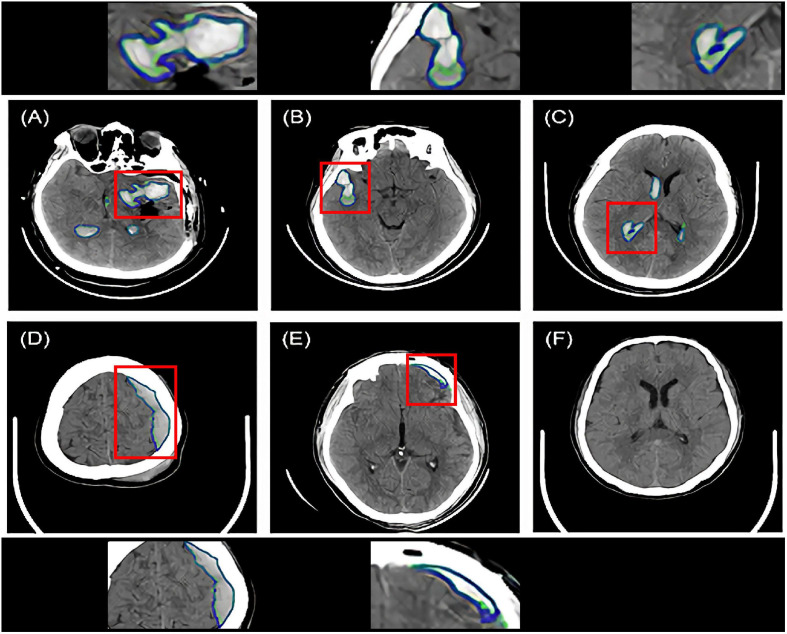
Intracranial hemorrhage segmentation examples of the Dense U-Net from six representative patients. The ground truth bleeding areas were contoured in green while the model segmentations were contoured in blue. Red boxes indicated zoomed-in regions of segmentation. **(A–C)** Three ICH segmentation examples. The patients had various hematoma shapes and positions. **(D)** One EDH segmentation example. **(E)** One SDH segmentation example. **(F)** No segmentation on the hematoma negative slice of the CT scan of the patient. ICH, intracerebral hemorrhage; EDH, extradural hemorrhage; SDH, subdural hemorrhage.

Figure 2 shows the segmentation examples of ICH, EDH, and SDH using the modified Dense U-Net. The ground truth segmentation (green) and the model predictions (blue) were overlaid on the same image. For each intracranial hemorrhage type, the segmentation Dices of the constructed DL model are displayed in Table 2.

**TABLE 2:** Dense U-Net segmentation Dice of intracranial hemorrhage.

Hemorrhage type	Dice
ICH	0.90 ± 0.06/0.86 ± 0.09*
EDH	0.88 ± 0.12
SDH	0.82 ± 0.16

ICH, intracerebral hemorrhage; EDH, extradural hemorrhage; SDH, subdural hemorrhage. *Denoted the segmentation Dice of ICH from the Hospital B.

### Statistical Analysis

For ICH from Hospital A, the difference mean between the ABC/2 method and the ground truth was 3.2 ml, while the difference standard deviation was 11.5 ml and the absolute difference mean was 7.3 ml. However, for ICH volume estimated by the deep framework, the difference mean was 1.3 ml, the standard deviation was 1.6 ml, and the absolute difference mean was 1.5 ml.

For ICH from Hospital B, the difference mean of the ABC/2 method was −2.1 ml while the difference standard deviation was 10.1 ml, and the absolute difference mean was 7.0 ml. For the DL model, the difference mean was −0.5 ml, with a standard deviation of 4.1 ml, and an absolute difference mean of 0.6 ml.

For EDH and SDH, only the hemorrhage volumes performed by the deep framework were analyzed because the ABC/2 method is not applicable for measuring the volumes of intracranial hemorrhage with irregular shapes. For EDH, the difference mean between the DL model and the ground truth was −0.3 ml, with a standard deviation of 1.4 ml, and an absolute mean of 1.1 ml. For SDH, the mean volume difference was −1.2 ml, with a standard deviation of 1.5 ml, and an absolute mean of 1.4 ml.

The Wilcoxon signed-rank test showed that the DL-based segmentation tended to systematically overestimate ICH volume by 1.0 ml for Hospital A (*p* < 0.001) and underestimated it by 0.4 ml for Hospital B (*p* < 0.001) compared with the ground truth. For EDH, there was no clear difference in hemorrhage volume estimation between the DL-based segmentation and the ground truth (*p* = 0.296). For SDH, the statistical test showed that the DL model underestimated the hemorrhage volume by 0.8 ml (*p* < 0.001). However, although the over- and underestimations were statistically significant, the biases were small and might not have clinical significance.

For data collected to evaluate the performance of the model on non-hemorrhagic cases, the DL model successfully identified 388 negative cases from 402 non-hemorrhagic cases with an accuracy of 96.5%.

**FIGURE 3:**
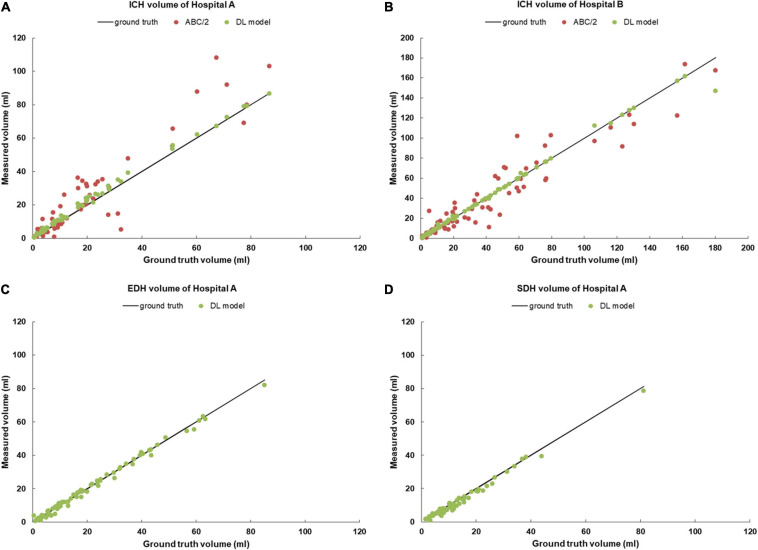
Scatter plots of hemorrhage volume measurements for **(A)** ICHs from the Hospital A; **(B)** ICHs from the Hospital B; **(C)** EDHs from the Hospital A; **(D)** SDHs from the Hospital A. ICHs, intracerebral hemorrhage cases; EDHs, extradural hemorrhage cases; SDHs, subdural hemorrhage cases.

In this study, we first revealed the consistency of the manual segmentations among the three radiologists and the reliability of the ground truth masks with high Dice (0.89–0.95) and ICC scores (0.985–0.997). Then, we demonstrated that the Dense U-Net framework was remarkably accurate in the automatic segmentation of intracranial hemorrhages, including ICH, EDH, and SDH, with high Dice scores for both internal (0.82–0.90) and external test sets (0.86). We further verified that compared with the ABC/2 method, ICH volume estimated by the DL model had a stronger correlation with the ground truth volume as reflected by ICC (0.998) and PCC (>0.996–0.998) (see Figure 3 and Table 3). Lastly, we assessed the model on a larger dataset containing non-hemorrhagic cases to verify its capability to segment lesions accurately without introducing more false positive results. These results indicated that the deep framework was more accurate than the ABC/2 method when quantifying the volume of large, complex-shaped intracranial hemorrhages.

**TABLE 3:** ICC and PCC of volume estimations.

Hemorrhage type	ICC		PCC	
	
	Model	ABC/2	Model	ABC/2
ICH	0.9978 0.9978*	0.9509 0.9780*	0.9979 0.9960*	0.9375 0.9576*
EDH	0.9983	–	0.9969	–
SDH	0.9882	–	0.9930	–

ICC, intra-class correlation coefficient; PCC, Pearson correlation coefficient; ICH, intracerebral hemorrhage; EDH, extradural hemorrhage; SDH, subdural hemorrhage. *Hospital B.

## Discussion

This study indicated that the performance achieved by the constructed Dense U-Net was comparable to the manual segmentation by the radiologists for brain hemorrhage and volume estimation. Additionally, the model was robust for a broad range of volume between 1 and 100 ml, and it could also be applied to various brain hemorrhage types in comparison with the previous findings reported (Scherer et al., 2016).

Moreover, the constructed Dense U-Net in this study used half of the dense units of the DenseNet reported previously (29 vs. 58 of DenseNet-121) (Huang et al., 2017). That is, our modification halved the model parameters, yet the model could still achieve high segmentation performance using fewer computational resources. With the current configuration and hardware conditions, the trained model could finish inferring one brain CT scan within approximately 5 s. The time-efficiency also enabled the possibility of the DL network to be applied to emergency settings, especially in the situation for brain hemorrhage patients.

According to the results on volume estimation, the ABC/2 method showed higher standard deviation and thus was more unstable compared to ground truth. The ABC/2 method uses the lengths of the principal axes of ellipsoid to assess its volume, yet the shape of lesions, i.e., the hemorrhagic regions, is much more irregular and therefore cannot be assessed accurately by this means consistently. Thus it is of great importance to find more widely applicable and accurate methods to estimate the hemorrhagic volume. Using deep learning algorithms to estimate hemorrhagic volume automatically is one potential approach and the performance of the proposed Dense U-Net in this study also verified its capabilities to segment and analyze hemorrhagic regions accurately and quickly. In order to further improve its performance, more possible influencing factors could be considered in future work, such as the location and shape of hemorrhagic regions.

Inter-institutional robustness of the model was checked by testing the model using internal and external test sets from different hospitals. The average Dice coefficient of the external test set (ICH from Hospital B) dropped about 4% compared with that of the internal test set (from Hospital A), which might be the reason that the external test datasets (from Hospital B) were not used for model training. For example, the two hospitals used different scanners as Hospital A mainly used scanners manufactured by GE and Siemens, while Hospital B mainly used scanners from GE and United Imaging Healthcare. Although only ICH scans were currently curated from Hospital B, the DL model still yielded excellent performance, indicating its strong robustness for the segmentation and quantification of intracranial hemorrhages. Furthermore, the good robustness of the Dense U-Net structure in different institutions could make its application significantly more widespread.

## Limitation

Though the small volume estimation difference of the segmentation-based method might not have any clinical significance (see the “Statistical Analysis” section of “Results”), it was interesting to note that this method tended to slightly overestimate the hemorrhage volume for ICH and systematically underestimate for SDH. More testing data might be needed to confirm this conclusion. Nonetheless, such a pattern might be related to how the model processed the edges of ICH and SDH. Since SDH was close to the skull, which also had high intensity on CT images, the model might tend to drop certain pixels of SDH at the interface. This might also be the reason for the relatively low Dice scores of SDH.

## Conclusion

This study demonstrated the high performance of a deep framework based on Dense U-Net for the automated segmentation and quantification of intracranial hemorrhages, including ICH, EDH, and SDH on non-contrast CT. Furthermore, the deep model also achieved strong robustness when tested on internal and external datasets from different hospitals. Moreover, the Dense U-Net utilized significantly fewer model parameters yet achieved accurate segmentation and precise volume quantification performance. With the high performance and time-efficiency, the model might potentially provide a promising tool to assist with treatment decisions for intracranial hemorrhages.

## Data Availability Statement

The datasets generated for this study are available on request to the corresponding author.

## Ethics Statement

The studies involving human participants were reviewed and approved by the Institutional Review Boards of Beijing Tiantan Hospital Affiliated to Capital Medical University and QingPu Branch of Zhongshan Hospital Affiliated to Fudan University. Written informed consent for participation was not required for this study in accordance with the national legislation and the institutional requirements.

## Author Contributions

JX, RZ, and JM conceived the study and participated in the literature search, study design, data collection, and data analysis. ZZ and CW completed the data analysis and interpretation. SW and GW completed the statistical analysis. RZ, HZ, and CX prepared the manuscript. YD edited the manuscript. All authors read and approved the final manuscript.

## Conflict of Interest

RZ, ZZ, QG, HZ, SW, YD, and CX were employed by Beijing Infervision Technology, Co., Ltd. The remaining authors declare that the research was conducted in the absence of any commercial or financial relationships that could be construed as a potential conflict of interest.

## Abbreviations

CT: computed tomography
DL: deep learning
EDH: extradural hemorrhage
ICH: intracerebral hemorrhage
ICC: intra-class correlation coefficients
IRB: Institutional Review Board
MRI: magnetic resonance imaging
PCC: Pearson correlation coefficient
SDH: subdural hemorrhage
SGD: stochastic gradient decay.

## References

[B1] Bottou L. (2010). “Large-scale machine learning with stochastic gradient descent,” in *Proceedings of COMPSTAT’2010*, (New York, NY: Springer), 177–186. 10.1007/978-3-7908-2604-3_16

[B2] Broderick J. P. Brott T. G. Duldner J. E. Tomsick T. Huster G. (1993). Volume of intracerebral hemorrhage. A powerful and easy-to-use predictor of 30-day mortality. *Stroke* 24 987–993. 10.1161/01.str.24.7.987 8322400

[B3] Chang P. Kuoy E. Grinband J. Weinberg B. Thompson M. Homo R. (2018). Hybrid 3D/2D convolutional neural network for hemorrhage evaluation on head CT. *Am. J. Neuroradiol.* 39 1609–1616. 10.3174/ajnr.a5742 30049723 PMC6128745

[B4] Chen T. Li M. Li Y. Lin M. Wang N. Wang M. (2015). *Mxnet: A flexible and efficient machine learning library for heterogeneous distributed systems.* arXiv preprint arXiv:1512.01274.

[B5] De Boer P.-T. Kroese D. P. Mannor S. Rubinstein R. Y. (2005). A tutorial on the cross-entropy method. *Ann. operations res.* 134 19–67. 10.1007/s10479-005-5724-z

[B6] Divani A. A. Majidi S. Luo X. Souslian F. G. Zhang J. Abosch A. (2011). The ABCs of accurate volumetric measurement of cerebral hematoma. *Stroke* 42 1569–1574. 10.1161/strokeaha.110.607861 21566231

[B7] Guan S. Khan A. Sikdar S. Chitnis P. (2019). Fully Dense UNet for 2D sparse photoacoustic tomography artifact removal. *IEEE J. Biomed. Health Inform.* 24:568–576. 10.1109/JBHI.2019.2912935 31021809

[B8] He K. Zhang X. Ren S. Sun J. (2015). “Delving deep into rectifiers: Surpassing human-level performance on imagenet classification,” in *Proceedings of the IEEE international conference on computer vision* (New Jersey, NY: Institute of Electrical and Electronics Engineers Inc). 1026–1034.

[B9] Hemphill J. C. Bonovich D. C. Besmertis L. Manley G. T. Johnston S. C. (2001). The ICH score. *Stroke* 32 891–897. 11283388 10.1161/01.str.32.4.891

[B10] Hemphill J. C. Lam A. (2017). Emergency neurological life support: intracerebral hemorrhage. *Neurocritical care* 27 89–101. 10.1007/s12028-017-0453-0 28913708

[B11] Huang G. Liu Z. Van Der Maaten L. Weinberger K. Q. (2017). “Densely connected convolutional networks,” in *Proceedings of the IEEE conference on computer vision and pattern recognition* (Honolulu: CVPR). 4700–4708.

[B12] Huttner H. B. Steiner T. Hartmann M. Köhrmann M. Juettler E. Mueller S. (2006). Comparison of ABC/2 estimation technique to computer-assisted planimetric analysis in warfarin-related intracerebral parenchymal hemorrhage. *Stroke* 37 404–408. 10.1161/01.str.0000198806.67472.5c 16373654

[B13] Kikinis R. Pieper S. D. Vosburgh K. G. (2014). “3D Slicer: a platform for subject-specific image analysis, visualization, and clinical support,” in *Intraoperative imaging and image-guided therapy* (New Jersey, NY: Springer), 277–289. 10.1007/978-1-4614-7657-3_19

[B14] Kothari R. U. Brott T. Broderick J. P. Barsan W. G. Sauerbeck L. R. Zuccarello M. (1996). The ABCs of measuring intracerebral hemorrhage volumes. *Stroke* 27 1304–1305. 10.1161/01.str.27.8.1304 8711791

[B15] Liu R. Li H. Hua Y. Keep R. F. Xiao J. Xi G. (2019). Early hemolysis within human intracerebral hematomas: an MRI study. *Trans. Stroke Res.* 10 52–56. 10.1007/s12975-018-0630-2 29766451

[B16] Mayer S. A. Rincon F. (2005). Treatment of intracerebral haemorrhage. *Lancet Neurol.* 4 662–672. 16168935 10.1016/S1474-4422(05)70195-2

[B17] Mendelow A. D. Gregson B. A. Fernandes H. M. Murray G. D. Teasdale G. M. Hope D. T. (2005). Early surgery versus initial conservative treatment in patients with spontaneous supratentorial intracerebral haematomas in the International Surgical Trial in Intracerebral Haemorrhage (STICH): a randomised trial. *Lancet* 365 387–397. 10.1016/s0140-6736(05)70233-6 15680453

[B18] Mendelow A. D. Gregson B. A. Rowan E. N. Murray G. D. Gholkar A. Mitchell P. M. (2013). Early surgery versus initial conservative treatment in patients with spontaneous supratentorial lobar intracerebral haematomas (STICH II): a randomised trial. *Lancet* 382 397–408. 10.1016/s0140-6736(13)60986-1 23726393 PMC3906609

[B19] Qureshi A. I. Mendelow A. D. Hanley D. F. (2009). Intracerebral haemorrhage. *Lancet* 373 1632–1644. 19427958 10.1016/S0140-6736(09)60371-8 PMC3138486

[B20] Rey D. Neuhäuser M. (2011). “Wilcoxon-signed-rank test,” in *International encyclopedia of statistical science*, ed. Lovric M. (Germany: Springer). 1658–1659.

[B21] Ronneberger O. Fischer P. Brox T. (2015). “U-net: Convolutional networks for biomedical image segmentation,” in *International Conference on Medical image computing and computer-assisted intervention* (New Jersey: Springer), 234–241.

[B22] Scherer M. Cordes J. Younsi A. Sahin Y.-A. Götz M. Möhlenbruch M. (2016). Development and validation of an automatic segmentation algorithm for quantification of intracerebral hemorrhage. *Stroke* 47 2776–2782. 10.1161/strokeaha.116.013779 27703089

[B23] Soffar H. M. H. (2012). *Comparative study between the outcome of duroplasty and dural snips in the management of acute subdural hematoma.* Ph. D. CU Theses, Cairo University, Egypt.

[B24] Srivastava N. Hinton G. Krizhevsky A. Sutskever I. Salakhutdinov R. (2014). Dropout: a simple way to prevent neural networks from overfitting. *J. Machine Learn. Res.* 15 1929–1958.

[B25] Xi G. Keep R. F. Hoff J. T. (2006). Mechanisms of brain injury after intracerebral haemorrhage. *Lancet Neurol.* 5 53–63. 10.1016/s1474-4422(05)70283-0 16361023

[B26] Yaghi S. Boehme A. K. Dibu J. Guerrero C. R. L. Ali S. Martin-Schild S. (2015). Treatment and outcome of thrombolysis-related hemorrhage: a multicenter retrospective study. *JAMA Neurol.* 72 1451–1457. 10.1001/jamaneurol.2015.2371 26501741 PMC4845894

